# The COVID-19 third wave in Myanmar following the military coup

**DOI:** 10.12688/f1000research.123450.2

**Published:** 2023-06-22

**Authors:** Spring Research Team

**Keywords:** COVID-19, Myanmar, mortality, third wave

## Abstract

**Background:** COVID-19 seriously hit Myanmar between June and August 2021, a few months after the military coup, though the first and second waves in 2020 were managed effectively by the government. People in Myanmar experienced serious consequences of the COVID-19 pandemic precipitated by the disorganized health system under the military junta. This study aimed to analyse the situation of COVID-19 occurrence and death proportions during its third wave in Myanmar.

**Methods:** An online survey was conducted using a Google form. People with the symptoms of COVID-19 and those who died from COVID-19 between June and August 2021 were eligible to participate. The Google form was extracted into an Excel datasheet and analysed using Stata v16.1.

**Results:** Among the 29,171 participants, 76.7% were over 30 years old and 56.4% were female. A majority of participants were from highly populated regions: Yangon (17,220; 59%) (Business capital), Mandalay (3,740; 12.8%) and Sagaing (1,546; 5.3%). Participants sought health care from telegram/other online services (34%), home care by health care providers (22%), private clinics (13%) and public hospitals run under the military junta (5%). Overall, 15% of participants died, of which, 72% occurred at home and 17% at public hospitals. Significantly higher proportions of deaths were seen among participants over 60 years than other age groups and males (p<0.001). Death proportions at different weeks from June to August 2021 ranged from 12.4% to 17.3%, much higher than the military junta’s reports. Overall, 25% of participants received oxygen therapy.

**Conclusions:** Death proportions in different weeks were consistently over 12%. The majority of participants received tele/online and home treatment services. Most deaths occurred at home. Findings indicated the high COVID-19 case fatality rates with limited access to public hospital care during the third wave. The data from this study suggest that the outcomes were adversely impacted by the military coup.

## Introduction

Coronavirus disease (COVID-19) is an infectious disease caused by the SARS-CoV-2 virus and its occurrence was first reported in China in December 2019. The virus spread rapidly inside China and to all parts of the globe. The World Health Organization announced the COVID-19 outbreak as a Public Health Emergency of International Concern on 30 ^th^ January 2020 and a pandemic on 11 ^th^ March 2021. As of the end of April 2022, there were over 513 million confirmed cases and over 6.2 million deaths worldwide. ^1^^,^^2^

Most COVID-19 cases suffer from mild illness and do not require hospitalization. ^3^ However, those with severe illness may need close monitoring and hospitalization. ^4^ Mortality is generally low. Contextual factors that may reduce the COVID-19 mortality included adequate level of health care resources like doctors, nurses, hospital beds, strong primary health care, low rate of health privatization and wide health coverage. ^5^ Public healthcare responses, efficiency of treatment, and COVID-19 variants may determine the COVID-19 severity and case fatality rate (CFR), ^6^ which is defined as the number of total deaths divided by the total confirmed cases, shown in per cent. As of July 2020, the global mean and median CFR were 3.31% and 2.19%, respectively, with the highest rate of 27% in Yemen. North Europe and North America also documented a high CFR of more than 10%. ^7^ CFR depends on the policies, responses, and efficiency of local healthcare systems, although its estimation has some flaws. ^6^

Myanmar had its first confirmed COVID-19 cases on 23 ^rd^ March 2020. Since then, the first wave of COVID-19 in Myanmar lasted for about three months, followed by a second wave in August 2020. Country level coordination, risk communication, and surveillance at point of entry were initiated in January 2020; before the WHO declaration of a global health emergency. In response to the pandemic, an increased number of COVID-19 testing centres, community quarantine sites, fever clinics, contact tracing measures, and guidelines were timely implemented. Confirmed cases were admitted to the COVID-19 designated centres and tertiary care hospitals, which provided comprehensive care to lower the adverse consequences and fatalities ( https://www.brookings.edu/blog/future-development/2020/12/01/myanmars-response-to-the-covid-19-pandemic/). ^8^ The “National COVID-19 Call Centre” was established to provide correct and timely COVID related health information to the general community https://dmr.gov.mm/officialannouncement/COVID_19CallCenter.pdf. The CFR during the first and second waves in Myanmar was 2.2%, which was similar to the global CFR. ^9^

The third wave of COVID-19 seriously hit Myanmar between June and August 2021, a few months after the military coup, which took place in February. People in Myanmar faced serious consequences of the COVID-19 pandemic, added to the disorganized health system impacted by the coup. Health workers in Myanmar have been oppressed by the military for providing medical care to injured protesting civilians and for participating in the Civil Disobedience Movement (CDM). The Insecurity Insight, Physicians for Human Rights (PHR), and John Hopkins University Center for Public Health and Human Rights (CPHHR) reported at least 252 attacks and threats against health workers and facilities during the first six months of the coup ( https://phr.org/news/at-least-252-reported-attacks-and-threats-to-health-care-in-myanmar-during-six-months-of-militarys-crackdown/)( https://phr.org/our-work/resources/violence-against-health-care-in-myanmar/). Consequently, people had limited access to healthcare services even when they had severe COVID-19 symptoms. Critical shortages of oxygen and medicines and movement restrictions at night due to curfew further compounded the situation ( https://phr.org/our-work/resources/violence-against-health-care-in-myanmar/) ( https://crisis24.garda.com/alerts/2021/07/myanmar-authorities-to-implement-stay-home-orders-nationwide-july-17-25-update-38).

To mitigate the COVID-19 third wave under the impact of military coup and inefficient health care system, Ministry of Health of the National Unity Government (NUG) implemented the online telehealth clinics for treating the COVID-19 patients and established the COVID-19 knowledge centre Facebook page to disseminate the correct and timely health messages to the community. ^10^ The NUG is the interim government formed by elected members of parliament, community leaders, representatives from Ethnic Resistance Organizations and CDM staff, and is recognized by the majority of Myanmar people ( https://www.iseas.edu.sg/articles-commentaries/iseas-perspective/2022-8-myanmars-national-unity-government-a-radical-arrangement-to-counteract-the-coup-by-moe-thuzar-and-htet-myet-min-tun/) ( https://aseanmp.org/2022/07/18/southeast-asian-mps-urge-asean-special-envoy-to-myanmar-to-meet-national-unity-government/).

Myanmar went through a COVID-19 third wave differently due to the difficult situation following the military coup. Therefore, this current study aimed to document the situation of the COVID-19 third wave in Myanmar, using an online survey.

## Methods

### Study design and sampling procedure

This is a cross-sectional descriptive study, based on an online survey using a Google form. The weblink to the Google form was made available to the public through a number of online outlets of Ministry of Health, National Unity Government of Myanmar such as Facebook page, Signal and Telegram.

### Data collection

Online data collection using a Google form was conducted between 1 ^st^ August 2021 and 30 ^th^ September 2021. The survey questionnaire was developed in the Myanmar language, after reviewing the literature and discussing with the relevant healthcare specialists. The questionnaire contained 13 questions and it took approximately 5 minutes to complete. Anyone who experienced COVID-19 symptoms themselves or family members staying in the same house, or knew someone who died from COVID-19 symptoms, between 1 ^st^ June 2021 and 31 ^st^ August 2021 (referring to the study period), could participate in the survey. There was no limitation or exclusion in age, sex, geographical areas of the participants. Efforts were made to minimize the sampling bias and to get the representative samples from the different geographical areas throughout the country by sharing the google form through the community networks from different regions.

Questions included in the assessment were background characteristics (age, sex, state/region), week of symptom onset, COVID-19 symptoms, COVID-19 testing and result, duration of illness, treatment seeking, oxygen therapy, outcome of disease, place of death (if the patient died).

### Data analysis

The Google form was extracted into an Excel datasheet, which was analysed using Stata v16.1. All the participants (N=29,171) were included in the analysis, reporting subgroups and missing values in detail. Prior calculation of sample size was not done in order to recruit the eligible participants from all regions across the country. The age variable was cleaned for typos and for mixed Myanmar and English entries. The multiple-response variables were re-coded into binary variables for each response for analysis. The categories of the remaining variables were pre-defined. Descriptive analysis was performed to report the participants’ characteristics, by geographical regions; the patterns of COVID-19 symptom presentations, by outcomes such as recovered or death; the use of a healthcare facility for treating COVID-19 symptoms, by oxygen treatment; numbers of symptomatic participants in relation to proportions of death over the study period; and the place of death of the deceased participants. The survey was structured with simple questions for the general public, aiming to report the broad picture of the COVID-19 burden in the population; therefore, it did not allow further complex analysis. No multivariable analysis was done and only bi-variate analyses were applied to describe the overall situation during the third wave.

### Ethical consideration

The first section of the Google data collection form provided the participants with the information on the survey and asked the participants’ consent to participate in the study. If they agreed to participate, they could continue answering the survey questions. The study strictly ensured the anonymity and confidentiality of the information. Questions focused only on the disease happening and did not include personal information except age, sex and geographic location. Ethical approval was not sought because of the non-functioning of almost all ethical boards in Myanmar due to the country’s political situation, and the low risk, anonymised nature of the data collection.

## Results

Table 1 shows the COVID-19 symptomatic participant’s characteristics and geographical distribution. A total of 29,171 symptomatic participants were included in the assessment and 77% were from the major cities such as Yangon, Mandalay and Sagaing. Others included the remaining states and regions in Myanmar. The community from all the administrative states and regions participated in the study showing a nationwide coverage of the survey. In general, 76.7% were over 30 years of age and, specifically, the 31–45 years group was most commonly reported (29%). The proportion of females (56.4%) exceeded males (42.8%). Nearly 76% of the participants developed COVID-19 symptoms in the month of July. Over 42% received the COVID-19 test and 90% of their results were positive. Nearly 44% recovered from all symptoms while 15.1% had died at the time of the survey.

**Table 1.** COVID-19 symptomatic participants’ characteristics and distribution in the country (June–August 2021).

	Total		Yangon		Mandalay		Sagaing		Other States & Regions	
	N	%	N	%	N	%	N	%	N	%
Total	29171	100.0	17220	100.0	3740	100.0	1546	100.0	6665	100.0
**Age ^**										
≤15y	648	2.25	363	2.13	83	2.25	33	2.15	169	2.57
16-30	6,071	21.05	3,440	20.2	805	21.79	356	23.21	1,470	22.33
31-45	8,402	29.13	5,075	29.8	1,116	30.21	403	26.27	1,808	27.46
46-60	6,723	23.31	3,838	22.54	868	23.5	393	25.62	1,624	24.67
>60	6,997	24.26	4,314	25.33	822	22.25	349	22.75	1,512	22.97
**Sex**										
Female	16,452	56.4	9,862	57.3	2,056	55.0	856	55.4	3,678	55.2
Male	12,491	42.8	7,238	42.0	1,660	44.4	676	43.7	2,917	43.8
Other	228	0.8	120	0.7	24	0.6	14	0.9	70	1.1
**Symptom onset**										
June	4,306	14.8	2,660	15.5	548	14.7	209	13.5	889	13.3
July	22,072	75.7	13,693	79.5	2,721	72.8	999	64.6	4,659	69.9
August	2,793	9.6	867	5.0	471	12.6	338	21.9	1,117	16.8
**COVID-19 test**										
Tested	12,300	42.2	6,752	39.2	2,076	55.5	757	49.0	2,715	40.7
Not tested	16,129	55.3	9,999	58.1	1,585	42.4	762	49.3	3,783	56.8
Don't know	742	2.5	469	2.7	79	2.1	27	1.8	167	2.5
**Test result among tested**										
Positive	11,075	90.0	5,980	88.6	1,924	92.7	702	92.7	2,469	90.9
Negative	1,112	9.0	701	10.4	142	6.8	50	6.6	219	8.1
Unknown	113	0.9	71	1.1	10	0.5	5	0.7	27	1.0
**Outcome**										
Recovered	12,764	43.8	7,604	44.2	1,685	45.1	650	42.0	2,825	42.4
Dead	4,404	15.1	2,631	15.3	570	15.2	248	16.0	955	14.3
Other *	12,003	41.2	6,985	40.6	1,485	39.7	648	41.9	2,885	43.3

Note: Sex = “others” are most likely due to that the respondents entered the data for multiple COVID-19 symptomatic observations and they didn’t know which one to select.

^ n=330 (1.13%) missing.

* Other group includes participants who still had COVID-19 symptoms or experienced complications of COVID-19 at the time of survey.

As mentioned in Table 2, the highest proportion of deaths was reported among the participants over 60 years (38.5%), which was followed by 18.7% between 46 to 60 years. Significant difference was detected in death proportions or case fatality rate according to age (p<0.001). A higher proportion of deaths was seen among males than females (20.1% *versus* 11.4%), which was significantly different (p<0.001). However, death proportions were not different according to the location.

**Table 2.** Associations of participants’ background characteristics and disease outcomes (Total N=29,171).

	Total	Alive		Dead		p value
	N	N	Row %	N	Row %	
Age *						<0.001
≤15 y	648	644	99.4	4	0.6	
16-30	6,071	6,003	98.9	68	1.1	
31-45	8,402	8,058	95.9	344	4.1	
46-60	6,723	5,464	81.3	1,259	18.7	
>60	6,997	4,306	61.5	2,691	38.5	
Sex						<0.001
Female	16,452	14,583	88.6	1,869	11.4	
Male	12,491	9,979	79.9	2,512	20.1	
Other	228	205	89.9	23	10.1	
Location						0.199
Yangon	17,220	14,589	84.7	2,631	15.3	
Mandalay	3,740	3,170	84.8	570	15.2	
Sagaing	1,546	1,298	84.0	248	16.0	
Other States & Regions	6,665	5,710	85.7	955	14.3

* n=330 missing (1.13%).

The pattern of COVID-19 symptom presentation relating to outcomes is described in Table 3. Overall, fever (84.1%), anosmia (68.1%), cough (65.6%), appetite loss (57.1%) and muscle ache (52.6%) were the most common symptoms and generally similar presentations were seen among those who recovered. In contrast, among the deceased, fever (82%), breathlessness (75.7%), fatigue (67.9%), cough (59.7%), and appetite loss (55.8%) were presented most commonly. Symptoms mostly lasted between 11 and 15 days (31%), followed by a longer duration of 16 days or more (30%). Over 25% of participants (15% recovered and 81% in the deceased group) received oxygen treatment during their illness.

**Table 3.** Pattern of COVID-19 symptom presentation relating to outcomes.

	Total		Alive		Dead	
	N	%	N	%	N	%
Total	29,171	100.0	24,767	100.0	4,404	100.0
Symptom ^						
Fever	24,539	84.1	20,926	84.5	3,613	82.0
Anosmia	19,857	68.1	18,431	74.4	1,426	32.4
Cough	19,147	65.6	16,519	66.7	2,628	59.7
Appetite loss	16,646	57.1	14,187	57.3	2,459	55.8
Muscle aches	15,342	52.6	13,542	54.7	1,800	40.9
Fatigue	15,307	52.5	12,318	49.7	2,989	67.9
Headache	10,820	37.1	9,852	39.8	968	22.0
Breathlessness	10,242	35.1	6,908	27.9	3,334	75.7
Runny nose	10,007	34.3	9,311	37.6	696	15.8
Sore throat	8,948	30.7	8,082	32.6	866	19.7
Diarrhoea/vomiting	7,797	26.7	6,649	26.8	1,148	26.1
Symptom duration						
3-5 days	3,641	12.5	3,071	12.4	570	12.9
6-10 days	7,425	25.5	6,094	24.6	1,331	30.2
11-15 days	9,030	31.0	7,814	31.6	1,216	27.6
16 days or more	8,707	29.8	7,540	30.4	1,167	26.5
Don't know	368	1.3	248	1.0	120	2.7
Oxygen treatment						
Yes	7,393	25.3	3,831	15.5	3,562	80.9
No	21,778	74.7	20,936	84.5	842	19.1

^ Multiple response variable.

Table 4 describes the use of healthcare facilities for treating COVID-19 symptoms, shown by oxygen treatment. The participants most commonly used online or telehealth services (34.3%) among different healthcare points. Nearly 22% invited the healthcare provider for a home visit and 13% sought healthcare from private clinics. Very few participants (4.8%) received healthcare from public hospitals. Among those who were treated with oxygen, 43% and 35% were treated with online or telehealth services, respectively. Only 14% were treated at public hospitals.

**Table 4.** Use of health care facility for treating COVID-19 symptoms, shown by oxygen treatment.

Health care facility ^	Total		Treated with oxygen *	
	N	%	N	%
Total	29,171	100.0	7,393	100.0
Online/tele	10,011	34.3	2,605	35.2
Not sought	6,976	23.9	400	5.4
Home visit by provider	6,381	21.9	3,211	43.4
Private clinic	3,790	13.0	951	12.9
Public hospital	1,394	4.8	1,052	14.2
Charity clinic	335	1.1	135	1.8
Out-patient dept.	335	1.1	139	1.9
Private hospital	303	1.0	255	3.4
Other	3,628	12.4	611	8.3

^ Multiple response variable.

* Oxygen treatment was not necessarily arranged by the treating health facility, except at hospitals, but self-arranged by buying or hiring oxygen cylinders or as such for individual use at home.

**Figure 1.**
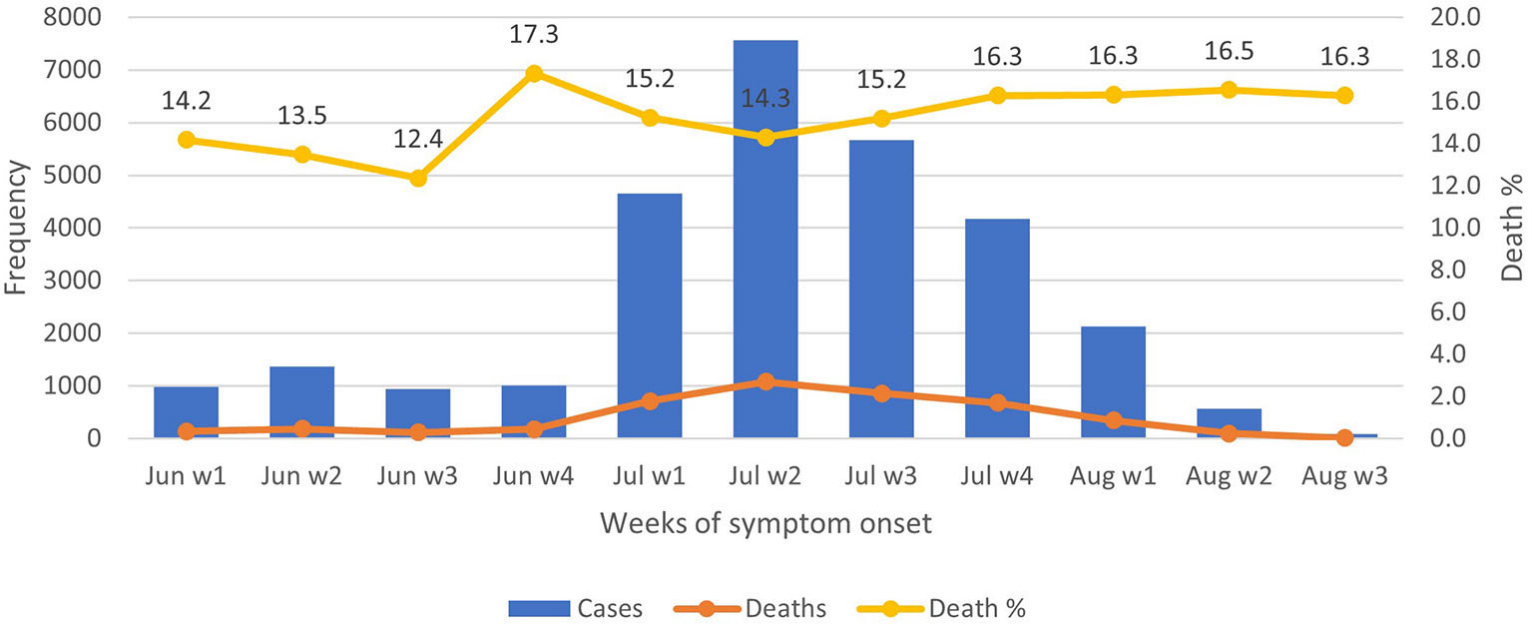
COVID-19 symptomatic observations and deaths from June to August 2021. Note: Aug w4 is omitted as it includes ‘0’.

**Figure 2.**
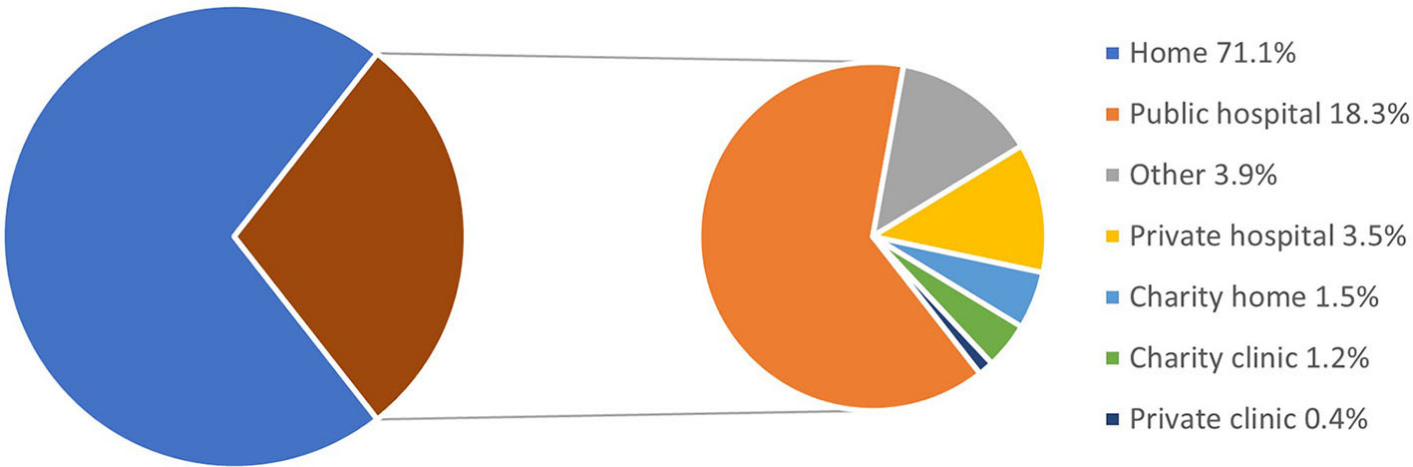
Places of death. Total N = 4,404.

Over the period of 1 ^st^ June 2021 to 31 ^st^ August 2021, the proportions of death ranged between 12.4% and 17.3%, with less variations throughout, despite the peak occurrence of COVID-19 in July week 2 ( Figure 1). The majority of deaths (72%) happened at home and 18.3%, at public hospitals ( Figure 2).

## Discussion

The current assessment documented the situation of COVID-19 occurrence and death proportions during its third wave in Myanmar, which took place under the impact of the military coup. The data indicated that the peak incidence happened during the 2 ^nd^ week of July 2021. Over half of the participants sought healthcare from Telegram/other online services and home care from healthcare providers. Significantly higher proportions of deaths were seen among older age groups (46 to 60 years and over 60 years) and males. Death proportions or CFR at different weeks were consistently over 12%. The majority of deaths occurred at home and only a few at public hospitals.

CFR is one informative epidemiologic tool that reflects the effectiveness of health policies, healthcare responses, and efficiency of health systems although its estimation has flaws. ^6^ In the current study, CFR was consistently high at above 12% over the study period, which was, in fact, much higher than the announcement made by the military junta (3.7%). ^11^ This study identified the overall death percent of 15.1%, while the global CFR remained 2.1%, according to the data as of July 2021. ^6^ During the first and second waves, in Myanmar, the CFR showed 2.2% (3,100 deaths among 140,600 COVID-19 confirmed cases), which was comparable to the global CFR. ^12^ This devastating increase in CFR in the third wave appears to be underpinned by the failed healthcare system following the coup.

COVID-19 deaths were different according to age group and gender. Based on the global data, death among males was 2.8% and that of females was 1.7%. With regards to the age group difference, the death rate was 3.6% in the 60–69 years old age group and 8% in the 70–79 years old group ( https://www.worldometers.info/coronavirus/coronavirus-age-sex-demographics/). Age group and gender differences in the current study were observed with much higher differences than that of the global data. Specifically, death among the older age group over 60 years was 38%, and that of males was 20%. This finding reflected an underlying vulnerability like old age had a greater impact of the limited healthcare access that leads to much higher mortality.

These adverse outcomes reflect the lack of essential healthcare for treating COVID-19 in Myanmar. This study identified that only a few COVID-19 symptomatic participants (4.8%) were treated at public hospitals with essential health facilities. Most participants, including those who received oxygen, were treated at places other than public hospitals. The findings highlighted that communities had limited access to the public hospitals run under the military junta. Before the coup, in 2015-2016, doctor population ratio was one doctor per 1,477 population which was below the WHO recommendation of one per 1,000 population. ^13^ Existing weakness in human resources in health was fueled by the attack of the military on the healthcare providers and health facilities, including the diversion of medical supplies to military use. Furthermore, arresting healthcare workers providing COVID-19 treatment outside of junta-run facilities worsened the condition ( https://phr.org/our-work/resources/violence-against-health-care-in-myanmar/).

About one-quarter of COVID-19 symptomatic participants in the current study received oxygen therapy. Medical oxygen is an essential medicine in the treatment of COVID-19 for cases with hypoxemia and is related to disease severity. ^14^^,^^15^ In a Chinese study, about 63% of COVID-19 patients under 65 years old admitted to hospital required oxygen therapy and mortality was 2.9% among the oxygen therapy patients. ^14^ In Myanmar, most COVID-19 cases were cared at home and given oxygen, when required, at home during the third wave. According to the news agencies, there were very high demands for oxygen in the community. ^16^ Worse, the military junta set restrictions on the sale and importation of oxygen cylinders during the period ( https://phr.org/our-work/resources/violence-against-health-care-in-myanmar/). Oxygen shortage was so severe that the family members of COVID-19 cases needed to queue for many hours to hire or get the oxygen cylinders filled. The crowded situation further precipitated the risk of disease transmission. Besides the oxygen shortage, rising pharmaceutical prices and shortage of medicines and other essential medical goods, including personal protective equipment (PPE), became a substantial strain on the Myanmar People. ^16^

Our study indicates that online/tele consultation was the most frequently used healthcare service for treating COVID-19. To address the healthcare needs of the community, the Ministry of Health of National Unity Government (NUG) had initiated telehealth free online clinics in June 2021. These telehealth online clinics cover 210 townships throughout the country within three months. Subsequently, COVID-19 specific Telegram channels were established in July. This service has provided consultation and treatment to more than 66,000 COVID-19 suspected and confirmed cases in 298 townships (90.3% of the total townships in Myanmar). ^10^ These telehealth clinics were efficient in providing healthcare services for mild-to-moderate COVID-19 cases during the third wave. It was reflected in the current study as over one-third of the COVID-19 symptomatic participants sought care from the online telehealth services.

There were both strengths and limitations in the current study. High participation from the community throughout the country making a large sample with nationwide coverage was a key strength of the study. Due to the nature of the online survey, clinical details of the participants could not be collected, neither could verification information, such as COVID-19 test results. At that time, people from Myanmar experienced COVID-19 third wave and because of limited testing capacity and situation following the coup, most patients were diagnosed and treated according to the symptoms. As the study was available online only and encouraged voluntary participation of the public, it was unable to estimate the disease prevalence or mortality in the population.

## Conclusion

The study documented the high COVID-19 case fatality rates with limited access to public hospital care during the third wave in Myanmar. The majority of participants received tele/online healthcare services and home treatment. CFR at different weeks were consistently much higher than the global data and most deaths occurred at home with little tertiary care. The data suggests that COVID-19 third wave severely hit Myanmar, with adverse outcome fueled by the military coup. However, the online Telehealth clinics operated by Ministry of Health, Myanmar National Unity Government, offered the alternative accessible solution for the certain population within the country.

## Data availability

Figshare: Underlying data for “The COVID-19 third wave in Myanmar following the military coup”, https://doi.org/10.6084/m9.figshare.20560017. ^17^

Data are available under the terms of the Creative Commons Attribution 4.0 International license (CC-BY 4.0).

## References

[1] Coronavirus: World Health Organization. Reference Source

[2] Coronavirus: World Health Organization. Reference Source

[3] Stokes EK Zambrano LD Anderson KN : Coronavirus disease case surveillance—United States, January 22–May 30, 2020. *MMWR Morb. Mortal. Wkly Rep.* 2019;69:759–765. 10.15585/mmwr.mm6924e2 Reference Source PMC7302472 32555134

[4] Centers for Disease Control and Prevention: Interim clinical guidance for management of patients with confirmed coronavirus disease (COVID-19). 2021. [Accessed 13 Dec 2021]. Reference Source

[5] Kapitsinis N : The underlying factors of excess mortality in 2020: a cross-country analysis of pre-pandemic healthcare conditions and strategies to cope with Covid-19. *BMC Health Serv. Res.* 2021;21(1):1197. 10.1186/s12913-021-07169-7 34736434 PMC8568489

[6] Abou Ghayda R Lee KH Han YJ : The global case fatality rate of coronavirus disease 2019 by continents and national income: A meta-analysis. *J. Med. Virol.* 2022;94(6):2402–2413. 10.1002/jmv.27610 35099819 PMC9015248

[7] Cao Y Hiyoshi A Montgomery S : COVID-19 case-fatality rate and demographic and socioeconomic influencers: worldwide spatial regression analysis based on country-level data. *BMJ Open.* 2020;10:e043560. 10.1136/bmjopen-2020-043560 33148769 PMC7640588

[8] Oo MM Tun NA Lin X : COVID-19 in Myanmar: Spread, actions and opportunities for peace and stability. *J. Glob. Health.* 2020;10(2):020374. 10.7189/jogh.10.020374 33110565 PMC7568915

[9] World Health Organization: WHO coronavirus disease (COVID-19) dashboard. 2020. [Accessed 9 Feb 2021]. Reference Source

[10] Ministry of Health, National Unity Government: One year activities by MOH, NUG. Reference Source

[11] Ministry of Health: *Coronavirus Disease 2019 (COVID-19), Surveillance Dashboard (Myanmar) Nay Pyi Taw.* Myanmar: Ministry of Health;2021. [Accessed 1 Dec 2021]. Reference Source

[12] World Health Organization: WHO coronavirus disease (COVID-19) dashboard. 2020. [Accessed 9 Feb 2021]. Reference Source

[13] Saw YM Than TM Thaung Y : Myanmar’s human resources for health: current situation and its challenges [published correction appears in Heliyon. 2019 Nov 01;5(10):e02737]. *Heliyon.* 2019;5(3):e01390. 10.1016/j.heliyon.2019.e01390 30976678 PMC6441837

[14] Ni YN Wang T Bm L : The independent factors associated with oxygen therapy in COVID-19 patients under 65 years old. *PLoS One.* 2021;16(1):e0245690. 10.1371/journal.pone.0245690 33481912 PMC7822310

[15] Yang X Yu Y Xu J : Clinical course and outcomes of critically ill patients with SARS-CoV-2 pneumonia in Wuhan, China: a single-centered, retrospective, observational study [published correction appears in Lancet Respir Med. 2020 Apr;8(4):e26]. *Lancet Respir. Med.* 2020;8(5):475–481. 10.1016/S2213-2600(20)30079-5 32105632 PMC7102538

[16] World Vision: Myanmar crisis response 2021: situation report 3. 5 August 2021. Reference Source

[17] Spring Research Team: Underlying data for “The COVID-19 third wave in Myanmar following the military coup” [data]. 2022. 10.6084/m9.figshare.20560017 PMC10576181 37841829

