# Supplementary material for: The COVID-19 third wave in Myanmar following the military coup
Source: F1000Res. 2023 Jun 22;11:1301. Originally published 2022 Nov 14. [Version 2] doi: 10.12688/f1000research.123450.2 (PMC10576181; doi:10.12688/f1000research.123450.2)
Supplement: Peer review history

# Reviewer report: Reviewer response for version 2

**DOI:** 10.5256/f1000research.151732.r180673

**Published:** 2023-10-13

**Authors:** Su Myat Han

**Affiliations:** 1 Faculty of Epidemiology and Population Health, London School of Hygiene and Tropical Medicine, London, UK; 2 School of Tropical Medicine and Global Health, Nagasaki University, Nagasaki, Japan

Thank you the authors for the revision. The revised version responded to all the reviewers comments and updated accordingly.

 I have no more comments or suggestion for the further revision.

Is the work clearly and accurately presented and does it cite the current literature?

Yes

If applicable, is the statistical analysis and its interpretation appropriate?

Yes

Are all the source data underlying the results available to ensure full reproducibility?

Yes

Is the study design appropriate and is the work technically sound?

Yes

Are the conclusions drawn adequately supported by the results?

Yes

Are sufficient details of methods and analysis provided to allow replication by others?

Yes

Reviewer Expertise:

universal health coverage, health services, humanitarian aids

I confirm that I have read this submission and believe that I have an appropriate level of expertise to confirm that it is of an acceptable scientific standard.

# Reviewer report: Reviewer response for version 2

**DOI:** 10.5256/f1000research.151732.r180674

**Published:** 2023-06-26

**Authors:** Nikos Kapitsinis

**Affiliations:** 1 University of Copenhagen, Copenhagen, Denmark

The authors have successfully revised the paper, that has significantly improved. Nice work

Is the work clearly and accurately presented and does it cite the current literature?

Yes

If applicable, is the statistical analysis and its interpretation appropriate?

Yes

Are all the source data underlying the results available to ensure full reproducibility?

Yes

Is the study design appropriate and is the work technically sound?

Yes

Are the conclusions drawn adequately supported by the results?

Yes

Are sufficient details of methods and analysis provided to allow replication by others?

Yes

Reviewer Expertise:

Health geography, Geographies of pandemics

I confirm that I have read this submission and believe that I have an appropriate level of expertise to confirm that it is of an acceptable scientific standard.

# Reviewer report: Reviewer response for version 1

**DOI:** 10.5256/f1000research.135555.r174658

**Published:** 2023-06-02

**Authors:** Nikos Kapitsinis

**Affiliations:** 1 University of Copenhagen, Copenhagen, Denmark

- In the introduction, second paragraph, I think it would very useful for the reader if the authors added and presented some socio-economic factors that affected the COVID-19 health implications. Several scholars have written about this. Illustrative studies that the authors could include are:

Kapitsinis, N. (2021) The underlying factors of excess mortality in 2020: a cross-country analysis of pre-pandemic healthcare conditions and strategies to cope with Covid-19. *BMC Health Services Research *21 *: *1197 ^1^.

 Pana T, Bhattacharya S, Gamble D, Pasdar Z, Szlachetka W, Perdomo-Lampignano J, et al. Country-level determinants of the severity of the first global wave of the COVID-19 pandemic: an ecological study. BMJ Open. 2021;11:e042034 ^2^.

- In the methods, the authors should explain more clearly and detailed how they distributed the online questionnaire, e.g. which online outlets they used. This will offer a more clear picture of methodology adopted.
- Moreover, the authors could present and describe whether the questionnaire included a question about the participant's status, i.e. whether he/she experienced COVID-19 or another person he/she knows. This is crucial to be clarified in the methodology section.
- The authors mention that over 42% of participants received the COVID-19 test. What about the others that reported they have been infected? Is there a question about how the participants realised/clarified that they had been infected by COVID-19? I.e. what about testing methods? The authors present it as one of the limitations of the study, in the discussion and they could elaborate a little more on this.
- I think the authors could present the questionnaire and its questions in the appendix.
- It would be useful if the authors presented some socio-economic data about Myanmar, in the discussion, and connect them with the capacity of the population to afford the private clinics and healthcare, but also the current status of public hospitals and healthcare in the country (number of doctors and medical beds per capita, for instance).
- They could also highlight the importance of the use of online consultation. What it means for the country, how it can help populations excluded from healthcare or quarantined and whether it could be more applicable in the future healthcare pathways.

Is the work clearly and accurately presented and does it cite the current literature?

Yes

If applicable, is the statistical analysis and its interpretation appropriate?

Yes

Are all the source data underlying the results available to ensure full reproducibility?

Yes

Is the study design appropriate and is the work technically sound?

Yes

Are the conclusions drawn adequately supported by the results?

Yes

Are sufficient details of methods and analysis provided to allow replication by others?

Yes

Reviewer Expertise:

Health geography, Geographies of pandemics

I confirm that I have read this submission and believe that I have an appropriate level of expertise to confirm that it is of an acceptable scientific standard, however I have significant reservations, as outlined above.

## References

[1] : The underlying factors of excess mortality in 2020: a cross-country analysis of pre-pandemic healthcare conditions and strategies to cope with Covid-19. *BMC Health Serv Res* .2021;21(1) : 10.1186/s12913-021-07169-7 1197 10.1186/s12913-021-07169-7 34736434PMC8568489

[2] : Country-level determinants of the severity of the first global wave of the COVID-19 pandemic: an ecological study. *BMJ Open* .2021;11(2) : 10.1136/bmjopen-2020-042034 e042034 10.1136/bmjopen-2020-042034 33536319PMC7868125

# Response

**Published:** 2023-06-17

**Authors:** Spring Research Team

**Affiliations:** Ministry of Health, Myanmar

**Comment 1:**

 In the introduction, second paragraph, I think it would very useful for the reader if the authors added and presented some socio-economic factors that affected the COVID-19 health implications. Several scholars have written about this. Illustrative studies that the authors could include are:

 Kapitsinis, N. (2021) The underlying factors of excess mortality in 2020: a cross-country analysis of pre-pandemic healthcare conditions and strategies to cope with Covid-19. BMC Health Services Research 21: 11971.

 Pana T, Bhattacharya S, Gamble D, Pasdar Z, Szlachetka W, Perdomo-Lampignano J, et al. Country-level determinants of the severity of the first global wave of the COVID-19 pandemic: an ecological study. BMJ Open. 2021;11:e0420342.

**Response 1:**

 Thanks for the suggestion. We added the socio-economic factors as you suggested in the second paragraph of the introduction as follows.

 “Contextual factors that may reduce the COVID-19 mortality included adequate level of health care resources like doctors, nurses, hospital beds, strong primary health care, low rate of health privatization and wide health coverage (Kapitsinis N, 2021).”

**Comment 2:**

 In the methods, the authors should explain more clearly and detailed how they distributed the online questionnaire, e.g. which online outlets they used. This will offer a more clear picture of methodology adopted.

  Moreover, the authors could present and describe whether the questionnaire included a question about the participant's status, i.e. whether he/she experienced COVID-19 or another person he/she knows. This is crucial to be clarified in the methodology section.

**Response 2:**

 Thanks for the comments. We added more explanation about the distribution of online questionnaire.

 “The weblink to the Google form was made available to the public through a number of online outlets of Ministry of Health, National Unity Government of Myanmar such as Facebook page, Signal and Telegram.”

 Regarding the respondent, anyone who experienced COVID-19 themselves or anyone in the family members answered the questionnaire. We edited accordingly in the data collection section.

**Comment 3:**

 The authors mention that over 42% of participants received the COVID-19 test. What about the others that reported they have been infected? Is there a question about how the participants realised/clarified that they had been infected by COVID-19? I.e. what about testing methods? The authors present it as one of the limitations of the study, in the discussion and they could elaborate a little more on this.  I think the authors could present the questionnaire and its questions in the appendix.

**Response 3:**

 Thanks for the comments. At that time, people from Myanmar had limited access to testing facility. We added more information on that issue in the limitation (last paragraph of the discussion section) as follows.

 “At that time, people from Myanmar experienced COVID-19 third wave and because of limited testing capacity and situation following the coup, most patients were diagnosed and treated according to the symptoms.”

 Regarding the questionnaire, we described the questions included in the 2 ^nd^ paragraph of the data collection section under the method. Journal does not allow to put the appendix.

**Comment 4:**

 It would be useful if the authors presented some socio-economic data about Myanmar, in the discussion, and connect them with the capacity of the population to afford the private clinics and healthcare, but also the current status of public hospitals and healthcare in the country (number of doctors and medical beds per capita, for instance).

 They could also highlight the importance of the use of online consultation. What it means for the country, how it can help populations excluded from healthcare or quarantined and whether it could be more applicable in the future healthcare pathways.

**Response 4:**

 Thanks. We added more information of some socio-economic data in the fourth paragraph of discussion section according to your suggestion as follows.

 “Before the coup, in 2015-2016, doctor population ratio was one doctor per 1,477 population which was below the WHO recommendation of one per 1,000 population (Saw YM, 2019). Existing weakness in human resources in health was fueled by the attack of the military on the healthcare providers and health facilities, including the diversion of medical supplies to military use.”

# Reviewer report: Reviewer response for version 1

**DOI:** 10.5256/f1000research.135555.r157852

**Published:** 2023-01-05

**Authors:** Su Myat Han

**Affiliations:** 1 Faculty of Epidemiology and Population Health, London School of Hygiene and Tropical Medicine, London, UK; 2 School of Tropical Medicine and Global Health, Nagasaki University, Nagasaki, Japan

**The COVID-19 third wave in Myanmar following the military coup**

 The current study added the value of the knowledge gap on the COVID-19 impact at the community level. The available research papers on the COVID-19 were mainly conducted at the hospital level and online people movement. This study particularly highlights the true burden of COVID-19 within the population. 

 Overall, this is a very good study, highlighting the disrupted health system and its impact on the health care sector response particularly at the pandemic situation of COVID-19. Below are the few comments and suggestions.

**Abstract**

**Background**: The first sentence of the background already concluding the severity of impact which is the main objective of the study. Background can be re-structured as “COVID-19 has disrupted health system of many countries including those developed nations. The impact is particularly worse in those resource limited settings including Myanmar. First and second waves in Myanmar during 2020 were effectively managed by the government. However, the momentum was disrupted due to the military coup in early 2021.  People in Myanmar experienced serious consequences of the COVID-19 pandemic precipitated by the disorganized health system under the military junta. This study aimed to analyze the situation of COVID-19 occurrence and death proportions during its third wave in Myanmar.”

**Conclusion:** The conclusion of " The data suggests that the outcomes were adversely impacted by the military coup." is very strong, but the results of the study does not directly refer to this outcome.

 I would suggest to improve the conclusion as:

 "The data suggests that COVID-19 third wave severely hit Myanmar, adverse outcome fueled by the military coup. However, the online Telehealth clinics operated by Ministry of Health (MoH), Myanmar National Unity Government (NUG), offered the alternative accessible solution for the certain population within the country."

**Introduction: **Well written and structured.

**Method: **Given the descriptive nature of the study (exploratory), the current method and data analysis are enough for the study. However, given the large number of participants, regression analysis can be performed to see the factors associated with outcomes (recovered/death/complications).  It is worthy to investigate risk of death (or) complications in association with cumulative symptoms (having two or more symptoms versus having only one by applying multilevel poisson regression model.

**Results**:

 Table (1): For those with outcome of death, who fill in the information in the google form. It would be great if the authors shared the survey google form (English translated version). For the COVID-19 test, what kinds of test the participants are taking (PCR, or rapid antigen test?). Is it included in the survey?

 Table (2): p-value column needs to be corrected (between the age and sex category).

 Table (3): OK , however, I noticed the variables are multiple response variables. It is worthy to investigate risk of death (or) complications in association with cumulative symptoms (having two or more symptoms versus having only one)  by applying multilevel poisson regression model.

 Table (4): It is better to recategorize the flow of the health care facility ( for example: others should be the last one).

**Discussion**: Well written and discussed accordingly with the results

**Conclusion:** The conclusion of " The data suggests that the outcomes were adversely impacted by the military coup." is very strong, but the results of the study does not directly refer to this outcome.

 I would suggest to improve the conclusion as:

 "The data suggests that COVID-19 third wave severely hit Myanmar, adverse outcome fueled by the military coup. However, the online Telehealth clinics operated by Ministry of Health (MoH), Myanmar National Unity Government (NUG), offered the alternative accessible solution for the certain population within the country."

Is the work clearly and accurately presented and does it cite the current literature?

Yes

If applicable, is the statistical analysis and its interpretation appropriate?

Yes

Are all the source data underlying the results available to ensure full reproducibility?

Yes

Is the study design appropriate and is the work technically sound?

Yes

Are the conclusions drawn adequately supported by the results?

Yes

Are sufficient details of methods and analysis provided to allow replication by others?

Yes

Reviewer Expertise:

universal health coverage, health services, humanitarian aids

I confirm that I have read this submission and believe that I have an appropriate level of expertise to confirm that it is of an acceptable scientific standard, however I have significant reservations, as outlined above.

# Response

**Published:** 2023-06-17

**Authors:** Spring Research Team

**Affiliations:** Ministry of Health, Myanmar

**Comment 1**

 The current study added the value of the knowledge gap on the COVID-19 impact at the community level. The available research papers on the COVID-19 were mainly conducted at the hospital level and online people movement. This study particularly highlights the true burden of COVID-19 within the population.

 Overall, this is a very good study, highlighting the disrupted health system and its impact on the health care sector response particularly at the pandemic situation of COVID-19. Below are the few comments and suggestions.

**Response 1:** Thanks so much for the comments.

**Comment 2:**

 Abstract

 Background: The first sentence of the background already concluding the severity of impact which is the main objective of the study. Background can be re-structured as “COVID-19 has disrupted health system of many countries including those developed nations. The impact is particularly worse in those resource limited settings including Myanmar. First and second waves in Myanmar during 2020 were effectively managed by the government. However, the momentum was disrupted due to the military coup in early 2021.  People in Myanmar experienced serious consequences of the COVID-19 pandemic precipitated by the disorganized health system under the military junta. This study aimed to analyze the situation of COVID-19 occurrence and death proportions during its third wave in Myanmar.”

**Response 2: **Thanks. We revise it based on your suggestion and word count limit as follows.

 “COVID-19 has disrupted health system of many countries, particularly worse in those resource limited settings including Myanmar. First and second waves in Myanmar during 2020 were effectively managed by the government. However, the momentum was disrupted due to the military coup in early 2021. This study aimed to analyse the situation of COVID-19 occurrence and death proportions during its third wave in Myanmar.”

**Comment 3:**

 Conclusion: The conclusion of "The data suggests that the outcomes were adversely impacted by the military coup." is very strong, but the results of the study does not directly refer to this outcome.

 I would suggest to improve the conclusion as:

 "The data suggests that COVID-19 third wave severely hit Myanmar, adverse outcome fueled by the military coup. However, the online Telehealth clinics operated by Ministry of Health (MoH), Myanmar National Unity Government (NUG), offered the alternative accessible solution for the certain population within the country."

**Response 3:** We revise it according to your suggestion.

**Comment 4:**

 Introduction: ****Well written and structured.

**Response 4:** Thanks

**Comment 5:**

 Method: ****Given the descriptive nature of the study (exploratory), the current method and data analysis are enough for the study. However, given the large number of participants, regression analysis can be performed to see the factors associated with outcomes (recovered/death/complications).  It is worthy to investigate risk of death (or) complications in association with cumulative symptoms (having two or more symptoms versus having only one by applying multilevel Poisson regression model.

**Response 5:**

 Thanks for your suggestion. However, as you also mentioned that the main objective of our study is to highlight the overall burden of the COVID-19 amidst the military coup, our current analysis addresses the main objective of the study.

**Comment 6:**

 Results:

 Table (1): For those with outcome of death, who fill in the information in the google form. It would be great if the authors shared the survey google form (English translated version). For the COVID-19 test, what kinds of test the participants are taking (PCR, or rapid antigen test?). Is it included in the survey?

 Table (2): p-value column needs to be corrected (between the age and sex category).

 Table (3): OK, however, I noticed the variables are multiple response variables. It is worthy to investigate risk of death (or) complications in association with cumulative symptoms (having two or more symptoms versus having only one) by applying multilevel poisson regression model.

 Table (4): It is better to recategorize the flow of the health care facility (for example: others should be the last one).

**Response 6:**

 Table (1): For those with outcome of death, respondent is immediate family member of a deceased who stays together in the same house. Among the respondents, only 42% had received COVID-19 test. Over half of them were diagnosed and treated by the symptoms at that time. For the type of COVID-19 test, we cannot ask whether the test is PCR or RDT since most of them are lay people.

 Table (3): Thanks for the suggestion. As we’ve explained above, the main objective of our study is to highlight the overall burden of the COVID-19 amidst the military coup, our current analysis addresses the main objective of the study.

 Table (4): Thanks. We recategorized the flow of health care facility as your suggestion.

**Comment 7:**

 Discussion: Well written and discussed accordingly with the results

**Response 7: **Thanks

**Comment 8:**

 Conclusion: The conclusion of " The data suggests that the outcomes were adversely impacted by the military coup." is very strong, but the results of the study does not directly refer to this outcome.

 I would suggest to improve the conclusion as:

 "The data suggests that COVID-19 third wave severely hit Myanmar, adverse outcome fueled by the military coup. However, the online Telehealth clinics operated by Ministry of Health (MoH), Myanmar National Unity Government (NUG), offered the alternative accessible solution for the certain population within the country."

**Response 8:**

 Thanks. We revise according to your suggestion.
